# HES6 promotes prostate cancer aggressiveness independently of Notch signalling

**DOI:** 10.1111/jcmm.12537

**Published:** 2015-04-12

**Authors:** Filipe L F Carvalho, Luigi Marchionni, Anuj Gupta, Basheer A Kummangal, Edward M Schaeffer, Ashley E Ross, David M Berman

**Affiliations:** a Department of Pathology, Johns Hopkins University School of MedicineBaltimore, MD, USA; b Department of Oncology, Johns Hopkins University School of MedicineBaltimore, MD, USA; c Brady Institute of Urology, Johns Hopkins University School of MedicineBaltimore, MD, USA; d Departments of Pathology and Molecular Medicine and Cancer Biology and Genetics, Cancer Research Institute, Queen’s UniversityKingston, ON, Canada

**Keywords:** Gleason score, HES6, NOTCH3, Notch pathway, prostate cancer

## Abstract

Notch signalling is implicated in the pathogenesis of a variety of cancers, but its role in prostate cancer is poorly understood. However, selected Notch pathway members are overrepresented in high-grade prostate cancers. We comprehensively profiled Notch pathway components in prostate cells and found prostate cancer-specific up-regulation of *NOTCH3* and *HES6*. Their expression was particularly high in androgen responsive lines. Up- and down-regulating Notch in these cells modulated expression of canonical Notch targets, HES1 and HEY1, which could also be induced by androgen. Surprisingly, androgen treatment also suppressed Notch receptor expression, suggesting that androgens can activate Notch target genes in a receptor-independent manner. Using a Notch-sensitive Recombination signal binding protein for immunoglobulin kappa J region (RBPJ) reporter assay, we found that basal levels of Notch signalling were significantly lower in prostate cancer cells compared to benign cells. Accordingly pharmacological Notch pathway blockade did not inhibit cancer cell growth or viability. In contrast to canonical Notch targets, HES6, a HES family member known to antagonize Notch signalling, was not regulated by Notch signalling, but relied instead on androgen levels, both in cultured cells and in human cancer tissues. When engineered into prostate cancer cells, reduced levels of HES6 resulted in reduced cancer cell invasion and clonogenic growth. By molecular profiling, we identified potential roles for HES6 in regulating hedgehog signalling, apoptosis and cell migration. Our results did not reveal any cell-autonomous roles for canonical Notch signalling in prostate cancer. However, the results do implicate HES6 as a promoter of prostate cancer progression.

## Introduction

Prostate-specific antigen (PSA) screening has vastly improved detection of prostate cancer at an early, curable stage 1,2. However, it has also resulted in overtreatment of many low-grade prostate cancers 3. This is because in early prostate tumour development, it is impossible to distinguish between slow-growing cancers that are relatively harmless and more aggressive tumours that could become life-threatening later on. In the absence of reliable prognostic tools, clinicians often feel compelled to provide costly and sometimes invasive treatments for early prostate cancers even though such efforts are likely to confer a survival benefit only in a minority of patients, *i.e*. men with aggressive tumours 4. Safely addressing overtreatment in prostate cancer will require a more sophisticated understanding of the molecular features that distinguish non-threatening prostate cancers from those that are potentially harmful.

Recent mRNA studies suggest that the expression patterns of certain genes associated with the Notch signalling pathway may aid in this distinction 5,6. The pathway is governed by four receptors, Notch1 through Notch4, and five ligands: Jagged 1 and 2 (JAG1 and JAG2) and Delta-like 1, 3 and 4 (DLL1, DLL3 and DLL4). When a ligand on the surface of a neighbouring cell binds to a Notch receptor, the receptor undergoes a conformational change that facilitates its cleavage by the ©-secretase complex. This cleavage releases the active form of the receptor—Notch intracellular domain (NICD)—into the cytoplasm. Cytoplasmic NICD translocates into the nucleus to interact with the DNA-binding transcriptional co-activator RBPJ. The resulting NICD/RBPJ complex activates Notch target genes, which include two families of transcriptional repressors: Hairy and enhancer of split (HES) and Hairy/enhancer of split related with YRPW motif (HEY) 7. Compared to their more indolent counterparts, aggressive prostate cancer cells have been reported to up-regulate JAG2, Notch3 and the HES family member HES6 5,6.

HES6 is of special interest because of its ambiguous status in the Notch signalling pathway and its apparent role in promoting cellular proliferation and migration in malignant glioma 8. Although HES6 bears strong homology to known Notch pathway targets, its regulation by Notch signalling has not been demonstrated. Nevertheless, HES6 can antagonize Notch signalling by interacting with HES1 9, reminiscent of other HES family members that participate in regulatory feedback loops.

Prior studies have shown that the Notch pathway plays important roles in normal prostate development. Notch signalling is critical for cell-fate decisions in many organs, including the prostate 7, and studies in mice have revealed that Notch ligands and receptors are expressed in both the basal and luminal layers comprising the prostate epithelium 10,11. Furthermore, the elimination of prostate cells expressing the Notch1 receptor blocks normal prostate development 10,11.

Notch also influences prostate epithelial cell responses to androgens. Luminal cells in the prostate express high androgen receptor (AR) levels and undergo apoptosis after androgen deprivation 12,13, whereas basal cells do not rely on androgen to survive and are able to regenerate prostate glands after castration once androgens are replenished 13. When prostate epithelial cells that express the Notch1 receptor are eliminated in castrated mice, the prostate fails to regrow even in the presence of androgens 13. This indicates the Notch pathway is crucial for androgen-induced prostate proliferative responses after castration.

The implication of the Notch pathway in the androgen responses of normal prostate cells is intriguing given that androgens play a prominent role in prostate cancer development. Prostate cancer cells originate as androgen-dependent epithelial cells with a luminal phenotype. Upon androgen deprivation, the cancer cells stop proliferating until they adapt to androgen-independent growth. Prostate cancer cell lines have been isolated from patients at different stages in this process, and can be classified as androgen responsive or androgen unresponsive. Whether the Notch pathway participates in prostate cancer cell responses to androgens is unknown.

The fact that aggressive prostate tumours induce heightened expression of a receptor, a ligand and a potential response gene all from the Notch signalling pathway implies that activation of the pathway may promote aggressiveness in prostate cancers. If so, tools and medicines designed to monitor and target Notch signalling could aid in the diagnosis and treatment of aggressive prostate tumours 14. We therefore conducted an intensive investigation of Notch pathway expression and function in benign and malignant prostate cell lines, confirming selected observations in human prostate tissues. Furthermore, we explored whether the pathway is involved in prostate cancer cell line responses to androgens. We also examined HES6 regulation and function to help clarify its status in the Notch pathway and to further explore its role in prostate cancer.

## Materials and methods

### Plasmids, virus and construct generation

HES6 (clone LIFESEQ7399489, Dharmacon, Lafayette, CO, USA) was subcloned into the lentiviral vector pNL-EGFP/CMV-WPREdU3 to establish overexpressing stable cell lines. shRNAs targeting HES6 mRNAs were obtained from the Expression Arrest-TRC shRNA Libraries (TRCN0000017828—target sequence CAGCCTGACCACAGCCCAAAT and TRCN0000017830—target sequence GCTGAACTGAGTCAGGCTCCT). Notch3 intracellular domain construct (gift from Dr. Nicolas Gaiano) was subcloned into pcDNA 3.1(-) (Invitrogen, Carlsbad, CA, USA). The vectors used in dual-luciferase reporter assay experiments were CBFRE-luc (Addgene #26897, Cambridge, MA, USA) and pGL2 (Promega, Madison, WI, USA).

### Cell lines and cell culture

All human cancer cell lines except VCaP and LnCaP96 were obtained from the American Type Culture Collection and maintained in RPMI 1640 (Life Technologies, Grand Island, NY, USA) supplemented with 10% foetal bovine serum (FBS) and Penicillin-Streptomycin (Invitrogen). The VCaP cell line was provided by Dr. William B. Isaacs (Brady Urological Institute, The Johns Hopkins University School of Medicine) and maintained in DMEM/10% FBS (Gibco). LnCaP96 cells were a gift from Dr. Alan K. Meeker (Brady Urological Institute, The Johns Hopkins University School of Medicine) and were generated by long-term culture of LnCaP in charcoal-stripped FBS. Prostate epithelial cells RWPE-1 and PrEC were cultured according to the vendor’s instructions. All cell line identities were confirmed by forensic identity analyses within 6 months of use. For the androgen withdrawal experiments, LnCaP cells were washed with charcoal-stripped FBS three times for 1 hr and incubated in 10% charcoal-stripped FBS-containing medium for 48 hrs before stimulation with 10 nM dihydrotestosterone (DHT).

### Real-time PCR analysis

Total RNA was isolated with RNeasy Mini kit (Qiagen, Valencia, CA, USA), and cDNA was synthesized with 1 μg of total RNA using high-capacity cDNA Reverse Transcription kit (Applied Biosystems, Foster City, CA, USA). cDNA was amplified on a StepOne Plus (Applied Biosystems) using TaqMan gene-specific oligonucleotide primers or custom-designed primers (Table S1). To determine the genes differentially expressed in cancer and benign cell lines, we designed Taqman gene plates comprising all known mammalian Notch pathway members and five housekeeping genes. Median-centred delta CT values were calculated for each gene, and those values were clustered by Euclidean distance and Ward linkage. The results are presented as a heat map.

### Immunoblotting

Protein lysates in NuPAGE buffer (Invitrogen) were separated by gradient 4–12% BisTris Gel and transferred to polyvinylidene difluoride membrane. Primary antibodies used in these experiments included rabbit monoclonal anti-Notch1 (D1E11; 1:1000; Cell Signaling, Danvers, MA, USA), rabbit polyclonal anti-cleaved Notch1 (Val1744; 1:1000; Cell Signaling), rabbit monoclonal anti-Notch2 (8A1; 1:1000; Cell Signaling), rabbit polyclonal anti-Notch3 (Pro2311; 1:1000; Cell Signaling), rabbit polyclonal anti-HES1 (ab71559; 1:1000; Abcam, Cambridge, MA, USA), rabbit polyclonal anti-HEY1 (ab22614; 1:500; Abcam), rabbit polyclonal anti-HES6 (ab66461; 1:1000; Abcam) and mouse monoclonal anti-glyceraldehyde-3-phosphate dehydrogenase (clone 6C5; 1:1000; Santa Cruz Biotechnology, Dallas, Tx, USA). The membrane was incubated sequentially with primary antibodies (overnight at 4°C), horseradish peroxidase-conjugated anti-rabbit or antimouse secondary antibodies, and chemiluminescent substrate (Thermo Fisher, Waltham, MA, USA) before exposure to film.

### Luciferase reporter assay

To determine endogenous Notch pathway activity in prostate cells, cells were plated in 24-well plates and transfected 24 hrs later using Lipofectamine2000 (Invitrogen) with pGL2 (control) or with CBFRE-luc. After 48 hrs, luciferase activity was determined using the Dual-Luciferase Reporter Assay system (Promega) and normalized to PrEC basal luminescence. All experiments were performed in triplicate.

### siRNA transient knockdown

Using Lipofectamine 2000 (Invitrogen), Notch3 siRNA (Table S2) transient transfections (final concentration of 10 nmol/l) were carried out according to the manufacturer’s instructions. Non-targeting siRNA was used as a control. Cells were lysed for Western blot analysis 24 hrs after siRNA transfection.

### DAPT treatment and IC50 assay

Twenty-four hours after seeding the cells, γ-secretase inhibitor *N*-[*N*-(3,5-Difluorophenacetyl)-l-alanyl]-*S*-phenylglycine t-butyl ester (DAPT) was added to 200 μl of growth media per well in final concentrations ranging from 1 nmol/l to 400 mmol/l. Viability was assayed (see below) after 96 hrs. The half maximal inhibitory concentration (IC50) of DAPT was calculated using GraphPad Prism software.

### Cell viability and proliferation assays

22Rv1 and LnCaP cells with stable shRNA HES6 knockdown or with PC3 stably overexpressing HES6 were seeded (500 cells per well) in 96-well black flat-bottom tissue culture plates. Cell viability and growth was determined using Alamar Blue vital dye (Invitrogen) according to the manufacturer’s instructions. Values were reported as the mean standard error of optical density for triplicate wells at each concentration and time-point.

### Immunohistochemistry and immunofluorescence

Immunohistochemistry and immunofluorescence staining and scoring were performed as described previously 14,15 using anti-Hes6 antibody (ab66461; 1:1000; Abcam). Areas of interest were outlined and HES6 staining intensity quantified using the TMAJ software package as described previously 16,17 The Mann–Whitney test used for the statistical analysis of HES6 staining intensity was measured by automated quantitative analysis.

### Androgen-deprived tissue in tissue microarray

We used a previously described tissue microarray comprised of formalin-fixed paraffin-embedded prostate cancer tissue from 55 patients who received androgen deprivation therapy prior to prostatectomy and from 12 untreated controls 15.

### Invasion assays

22Rv1 or LnCaP cells infected with control or shHES6 lentiviruses (see plasmids and viruses above) were seeded at 7.5 × 104 cells/well. PC3 cells infected with control or HES6 overexpression lentiviruses were seeded at 5 × 104 cells/well. The assay was performed in triplicate and in two independent experiments as previously described 18. The Student’s *t*-test was used to evaluate significant differences, and statistical significance was defined as *P* < 0.05.

### Colony-forming efficiency assay

Colony-forming efficiency assays were conducted as described earlier 19. All visible colonies were counted, and the results were presented as the total number of colonies from two individual experiments.

### Gene expression arrays and data analysis

Differential gene expression analysis was performed as previously described 6,20,21 using statistical packages from the R/Bioconductor project 22,23. Briefly, gene expression was measured on the Illumina HT-12 v4 whole genome gene expression microarray. For each individual microarray feature, a generalized linear model was fit to estimate expression differences between groups. Moderated t-statistics were obtained by empirical Bayesian shrinkage of log2 fold-change standard errors 24, and adjustment for multiple testing was obtained using the Benjamini and Hochberg method 25. Gene annotation for the microarray used in this study was obtained from the corresponding R/Bioconductor metadata packages.

## Results

### Notch pathway components in prostate carcinogenesis

**Figure 1:**
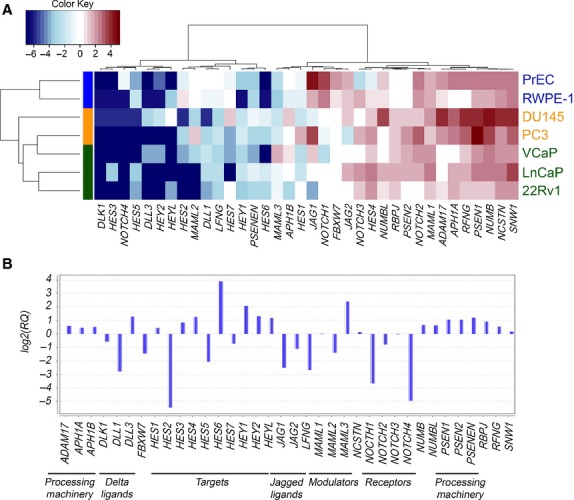
Notch pathway members’ expression in prostate cells. (A) Heat map showing qPCR mRNA transcript expression of Notch pathway members across prostate cells lines. Colour bars at the left of the heat map represent groups of cells with similar phenotypes: blue—benign cells; yellow—androgen-independent cancer cell lines; green—androgen responsive cancer cell lines. Hierarchical clustering (thin black lines at left) shows the gene expression patterns clearly distinguish benign from cancer cells. (B) qRT-PCR analysis showing average relative expression of Notch pathway members in cancer cells relative to average levels in benign cell lines. A log2 fold increase in the *y*-axis represents an enrichment of the genes in cancer cells, and negative values correspond to genes down-regulated in cancer cells. HES6 was the most induced gene in cancer cells. In contrast, canonical Notch signalling components, including DLL1, JAG1, NOTCH1 and NOTCH2, were down-regulated in cancer cells.

We investigated the expression of the 36 known and presumed Notch pathway components in a panel of commonly studied prostate cell lines. Two of these lines, RWPE-1 and PrEC, are benign and have been reported to show relatively luminal and basal epithelial phenotypes, respectively 26,27. To our knowledge these are the only prostate cell lines that are not are able to form colonies in soft agar or tumours when injected into immunodeficient mice. On the contrary, subcell lines derived from RWPE-1, such as RWPE-2 and WPE1-NA22, are able to form colonies in soft agar and tumours in immunodeficient mice 26. The luminal phenotype in RWPE-1 includes expression of AR and of cytokeratins 8 and 18 26. PrEC exhibits a basal epithelial cell phenotype, including the absence of AR and presence of basal-cell markers—p63 and cytokeratins 5 and 14 27. Interestingly, engineered overexpression of AR in immortalized PrEC induced a luminal cell phenotype with PSA expression, cell growth stimulated by androgens and formation of cribriform tumour nodules when implanted into the prostate of immunodeficient mice 28. Of the five cancer cell lines studied, two lines, DU145 and PC3, are androgen unresponsive and three, 22Rv1, LnCaP and VCaP, are androgen responsive 29–31. We performed quantitative TaqMan PCR (qPCR) assays on cDNA prepared from these prostate cell lines, and the results revealed Notch gene expression patterns that organized the cell lines clearly into distinct benign and cancer clusters (Fig.1A). A comparison of gene expression in benign *versus* malignant prostate cell lines identified HES6 as the most differentially expressed gene: HES6 transcripts were virtually undetectable in benign cells (Fig.1A) but yielded 4-fold higher transcript levels in cancer cells (Figs.1B). Other Notch targets (HEY1, HEY2 and HES4) also exhibited increased transcript levels in cancer cells compared to benign prostate cells (Fig.1B), though the differences in expression were less dramatic than that observed for HES6. In contrast, transcripts encoding several canonical Notch signalling components, including DLL1, JAG1, NOTCH1 and HES2, were down-regulated in cancer cells (Fig.1A and B).

### Notch signalling components in prostate cancer cell lines

**Figure 2:**
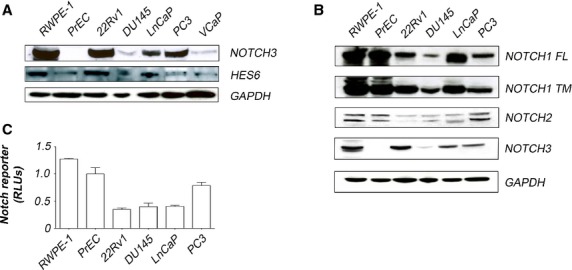
Dynamics of Notch signalling in prostate cells. (A) Immunoblot showing correlation of NOTCH3 and HES6 protein levels. Note that basal NOTCH3 and HES6 protein levels are globally higher in cancer cells than in benign cells, confirming the mRNA expression pattern presented (Fig.1A). (B) Immunoblot with antibodies against NOTCH1, NOTCH2 and NOTCH3 receptor proteins shows significantly higher levels of receptor proteins in benign cells than in cancer cells. FL: full length NOTCH1; TM: transmembrane NOTCH1. (C) Notch signalling levels as indicated by RBPJ-luciferase reporter. Note higher RBPJ promoter activity in benign cell lines, consistent with their higher expression levels of Notch receptors. RLUs: Relative Luminescence Units.

Previous studies reported increased Notch3 levels in aggressive prostate cancers 5,6. We therefore performed additional studies to further investigate Notch receptor expression in cancer, this time focusing on protein levels. Interestingly, the two benign prostate cell lines, RWPE-1 and PrEC, differed dramatically in terms of Notch3 protein levels. Indeed, of all the cell lines studied, RWPE-1 exhibited the highest levels of Notch3 protein, whereas PrEC had the lowest (Fig.2A and Fig. S1). When we compared Notch3 levels between benign and malignant cell lines, this phenomenon greatly influenced the outcome: If RWPE-1 was included in the analysis, we observed an overall lower expression of Notch3 in cancer. However, if RWPE-1 is excluded, Notch3 becomes the second most overexpressed gene in prostate cancer after HES6 (Fig. S2). Of further interest, cell lines with high Notch3 expression also exhibited increased HES6 expression. The expression of both HES6 and Notch3 was particularly elevated in three of four androgen responsive prostate cell lines (RWPE-1, 22Rv1 and LnCaP, but not VCaP) compared to androgen-independent lines (DU-145 and PC3) (Fig.2A). These results prompted us to explore whether Notch3 and/or androgens regulate HES6.

### Notch signalling dynamics in prostate cancer

Few previous studies have investigated the dynamics of Notch signalling in prostate cells. To address this, we used a variety of assays to measure the effects of genetic and pharmacologic manipulation on Notch pathway activity and cell viability in benign and malignant prostate cells. At baseline, benign prostate cells showed significantly higher levels of Notch receptor expression and greater responsiveness to Notch signalling than cancer cells. Specifically, benign prostate cells had the highest levels of Notch1 protein (Fig.2B) and higher levels of Notch2 protein than three of four cancer lines (Fig.2B). Likewise, introduction of a Notch-dependent luciferase reporter 32 revealed higher levels of baseline Notch signalling in benign cell lines than in cancer lines (Fig.2C). Thus, relative to benign prostate epithelial cells, prostate cancer cells appear to suppress constitutive expression and activation of the Notch pathway.

### Contributions of Notch3 receptor to Notch signalling and HES6 expression

Notch receptors differ from one another significantly in their biological effects 33 such that one Notch receptor may have a much greater biological impact on a given cell population than another. Our results show that Notch3 is the most highly induced Notch receptor in prostate cancer (Fig.2A)—a finding that suggests this particular receptor may perform an important function in this context. Our results also show that HES6 is the most highly induced putative Notch response gene in prostate cancer cells (Fig.2A). Furthermore, HES6 expression correlates closely with that of Notch3, consistent with the notion that HES6 is a target of the Notch signalling pathway. We therefore investigated whether Notch3 could induce HES6 expression.

**Figure 3:**
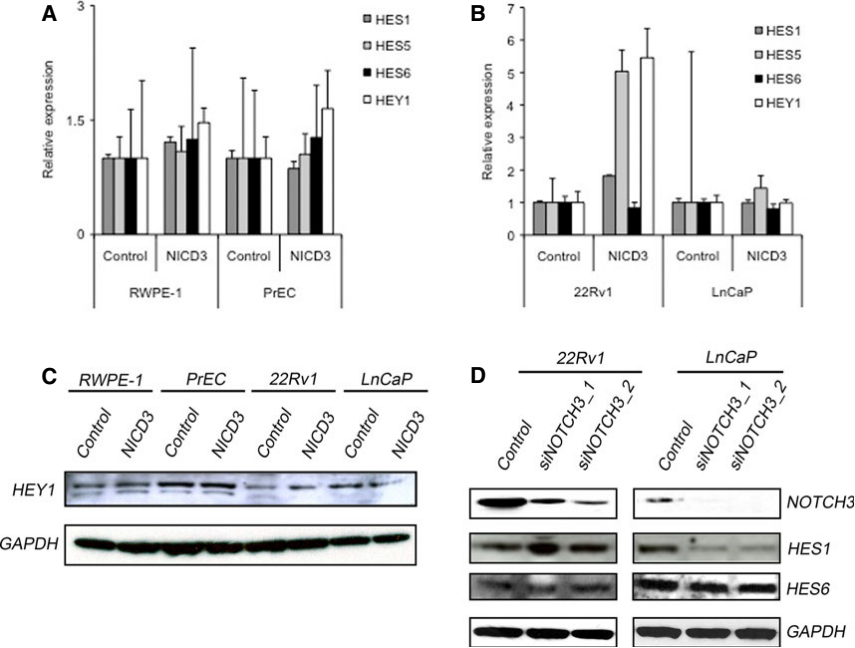
Notch signalling in prostate cells affects HES1, HES5 and HEY1 levels, but not HES6 levels. (A and B) qPCR analysis of HEY1, HES1, HES5 and HES6 levels after engineered NICD3 expression in benign and androgen responsive cancer cell lines. Note lack of response in benign lines *versus* up-regulation of HES1, HES5 and HEY1 in 22Rv1 cells. (C) Immunoblot with antibodies against HEY1 confirms that NICD3 expression in 22Rv1 induces HEY1expression. (D) Immunoblot shows knockdown of NOTCH3 levels with 2 independent siRNAs, resulting in suppression of HES1 in LnCaP cells but not in 222RV1. Note that HES6 protein levels were unaffected by NOTCH3 knockdown in both cell lines.

We introduced constitutively active Notch3 intracellular domain (NICD3) into benign (RWPE-1 and PrEC) and malignant (22Rv1 and LnCaP) prostate cells (Fig. S3) and used qPCR to measure the expression of known Notch target genes. Benign RWPE-1 and PrEC lines showed no significant induction of Notch targets by NICD3 (Fig.3A). Most cancer cell lines also showed only modest responses to NICD3. However, 22Rv1 cells responded with a 50% induction of HES1 and a 5-fold induction of HEY1 and HES5 (Fig.3B). Notably, HES6 was not induced by NICD3 in any of the cell lines we tested. These results indicate that some prostate cancer cell lines respond to Notch signalling, and they identify HES1, HEY1 and HES5 as potential Notch response genes in prostate cancer. Notably, along with the expression data reported above, these results also indicate that HES6 levels do not typically respond to Notch signalling in prostate cancer.

To confirm that HES6 is not under Notch3 control, we used two separate siRNAs to knock down Notch3 expression in 22Rv1 and LnCaP (*i.e*. the cancer lines with the highest Notch3 and HES6 levels) and measured the protein levels of both HES6 and HES1, a known Notch3 target 34. Upon NOTCH3 knockdown, levels of HES1 remained steady in 22Rv1 cells but dropped in LnCaP cells (Fig.3D), lending further support to the idea that HES1 responds to Notch signalling in prostate cancer cells. The findings further suggest that 22Rv1 has redundant mechanisms for supporting Notch signalling and/or HES1 expression that are lacking in LnCaP cells. In contrast to HES1, HES6 expression appeared to be independent of Notch signalling. Neither engineered expression of NICD3 (Fig.3B and C) nor Notch3 knockdown by siRNA (Fig.3D) affected HES6 levels. Although HES6 may interact with Notch signalling components, these results, along with expression data above, suggest that factors other than Notch signalling regulate HES6 expression in prostate cells.

### Prostate cancer cells are resistant to ©-secretase inhibition

**Figure 4:**
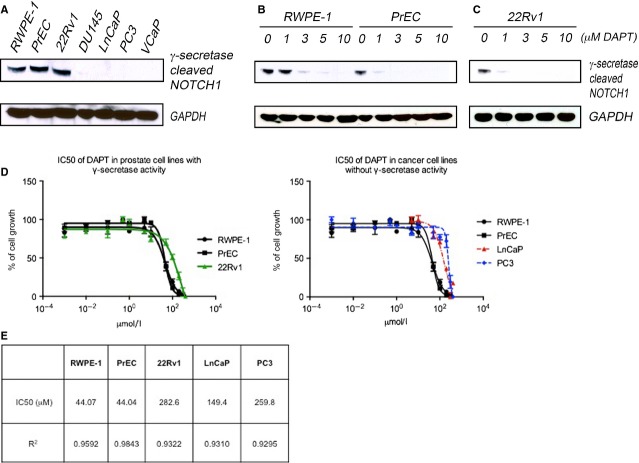
Prostate cancer cells are resistant to ©-secretase inhibition. (A) Immunoblot shows ©-secretase-cleaved NICD1 in two benign cell lines, PrEC and RWPE-1, and in the 22Rv1 cancer cell line. (B and C) Immunoblot showing the levels of NICD1 protein after treatment for 48 hrs with the indicated concentrations of the ©-secretase inhibitor DAPT. (D) Effects of DAPT on benign and cancer cell proliferation following incubation with increasing concentrations of DAPT. Error bars, mean ± SEM of triplicates. (E) Table listing IC50 for DAPT. Note that IC50 concentrations were above 40 μM—much higher concentrations than those required to inhibit ©-secretase.

Further examining the biological relevance of Notch signalling in prostate cells, we tested the effects of Notch pathway inhibition on prostate cell growth and viability. To do this, we treated prostate cell lines with the ©-secretase inhibitor DAPT and monitored cleavage of the Notch1 protein into its active moiety, NICD1. The two benign cell lines, PrEC and RWPE-1, exhibited high baseline levels of NICD1, but among five different cancer lines, only one 22Rv1 had detectable NICD1 levels (Fig.4A). Treatment of PrEC, RWPE-1 and 22Rv1 cells with 3 µM DAPT was sufficient to block ©-secretase activity and eliminate NICD1 protein levels (Fig.4B and C).

To examine the biological significance of this blockade, we treated both benign cell lines and three cancer lines (22Rv1, LnCaP and PC3) with DAPT and monitored cell growth. In all lines, a drop in cell viability required DAPT concentrations that were 30–40 times higher than those required to suppress NICD1 levels (Fig.4D and E). This is most likely an off-target, toxic effect rather than a biologically meaningful response to Notch inhibition. Thus, the results suggest Notch signalling is not required for survival or growth of benign or malignant prostate epithelial cells.

### Androgens down-regulate Notch receptors while up-regulating HES/HEY family members

Since androgen responsive cell lines showed distinctive expression patterns of Notch pathway components (Fig.1A), we investigated how androgens affect the expression of Notch receptors and target genes in prostate cancer cell cultures. After stimulating LnCaP cells with DHT, we collected RNA at time-points up to 48 hrs after initiating DHT treatment. We then used qPCR to measure the expression of Notch pathway components and the androgen responsive gene *PSA* 35. We observed robust responses to androgen as measured by *PSA* transcript levels (Fig. S4). *NOTCH4* was undetectably low regardless of androgen treatment, but all other Notch pathway components exhibited androgen responses.

**Figure 5:**
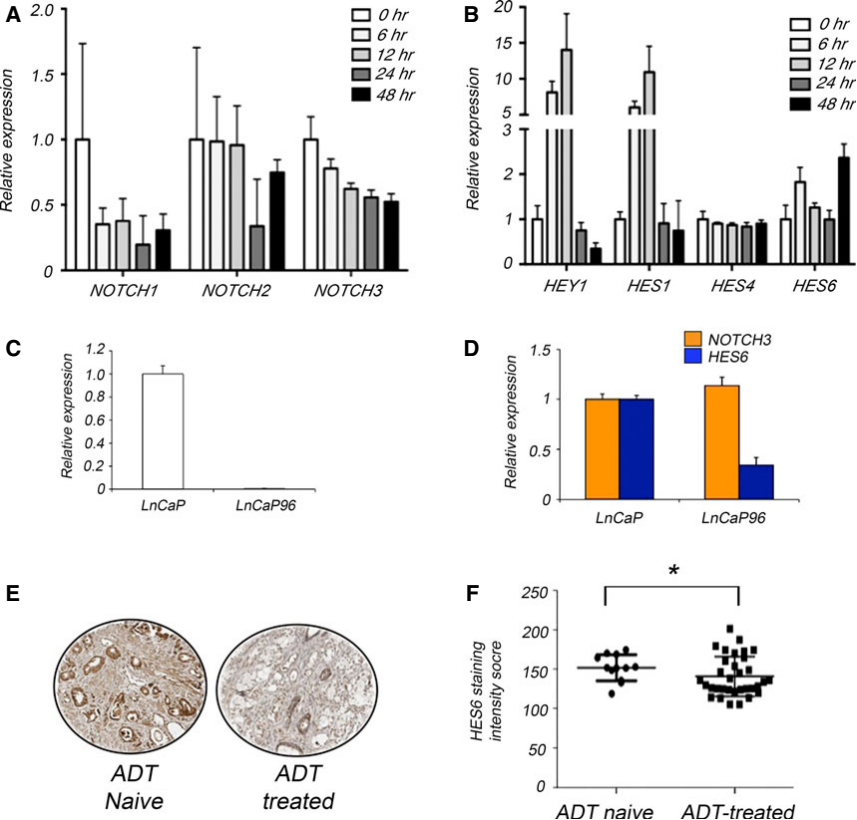
Androgens down-regulate Notch receptors and up-regulate HES/HEY family members. (A and B) Graphs showing qPCR analysis of mRNA transcripts encoding Notch receptors (A) and HES/HEY family members (B) in androgen-treated (10 nM DHT) LnCaP cells. Note decrease of receptor transcripts and increase of HEY1, HES1 and HES6. Error bars, mean ± SEM of three technical triplicates. (C and D) Graphs showing qPCR analysis of transcripts encoding the androgen response gene *PSA* in androgen-dependent LnCaP cells and in the sub-line LnCaP96, which was adapted to androgen-independent growth. Error bars, mean ± SEM of three technical triplicates. (E) Photomicrographs showing examples of immunohistochemical staining with antibodies against HES6 in untreated and androgen-deprived prostate cancer glands. (F) Scatter dot-plot showing immunohistochemical staining in prostate cancer tissues taken from patients who received androgen deprivation therapy (ADT) *versus* ADT-naïve prostate cancers (*P = 0.028, Mann–Whitney test).

Surprisingly, we found that DHT modulated Notch receptors and targets in opposite directions: Expression of *NOTCH1*, *NOTCH2* and *NOTCH3* receptors diminished after the initiation of DHT treatment, whereas levels of *HES1*, *HES6* and *HEY1* increased (Fig.5A and B). *HES1* and *HEY1* were markedly induced in the first 12 hrs of DHT treatment, though the effect subsided at later time-points. In contrast, *HES6* expression did not increase as quickly or dramatically as that of *HES1* and *HEY1*, but the response was more durable (Fig.5B). These results indicate that HES/HEY family members respond to androgen stimulation independently of canonical Notch signalling, and the response occurs in two phases: an initial phase characterized by rapid, transient induction of the Notch targets HEY1 and HES1, and a second phase marked by slow, sustained induction of HES6.

To further investigate the long-term effects of androgen withdrawal, we assayed the expression of HES/HEY family members in LnCaP96, an LnCaP sub-line adapted to androgen-independent growth 36. In LnCaP96, *PSA* transcripts were undetectable, consistent with the cell line’s androgen-independence, whereas *PSA* was highly expressed in the androgen-dependent parental LnCaP cells (Fig.5C). *NOTCH3* levels were the same in LnCaP96 and LnCaP cells. However, *HES6* levels were significantly reduced in LnCaP96 compared to LnCaP (Fig.5D). In agreement with these *in vitro* results, immunohistochemical assays revealed significantly reduced HES6 protein levels in cancers from men who had undergone long-term androgen deprivation therapy (ADT) compared to ADT-naïve cancers (Fig.5E and F). These results suggest that androgens induce HES/HEY family members, including HES6, through a Notch-independent mechanism.

### HES6 contributes to invasiveness and clonogenic growth

As shown by qPCR array, *HES6* transcripts were approximately fourfold enriched in prostate cancer cells compared to benign prostate cells (Fig.1B). In previous study, immunohistochemical analysis of HES6 mRNA and protein in human clinical samples 37 confirmed that HES6 was up-regulated in cancer and further demonstrated that strong nuclear HES6 protein expression increased as a function of Gleason grade, a potent indicator of metastatic potential in prostate cancer. The latter results imply HES6 may help promote prostate tumour aggressiveness.

**Figure 6:**
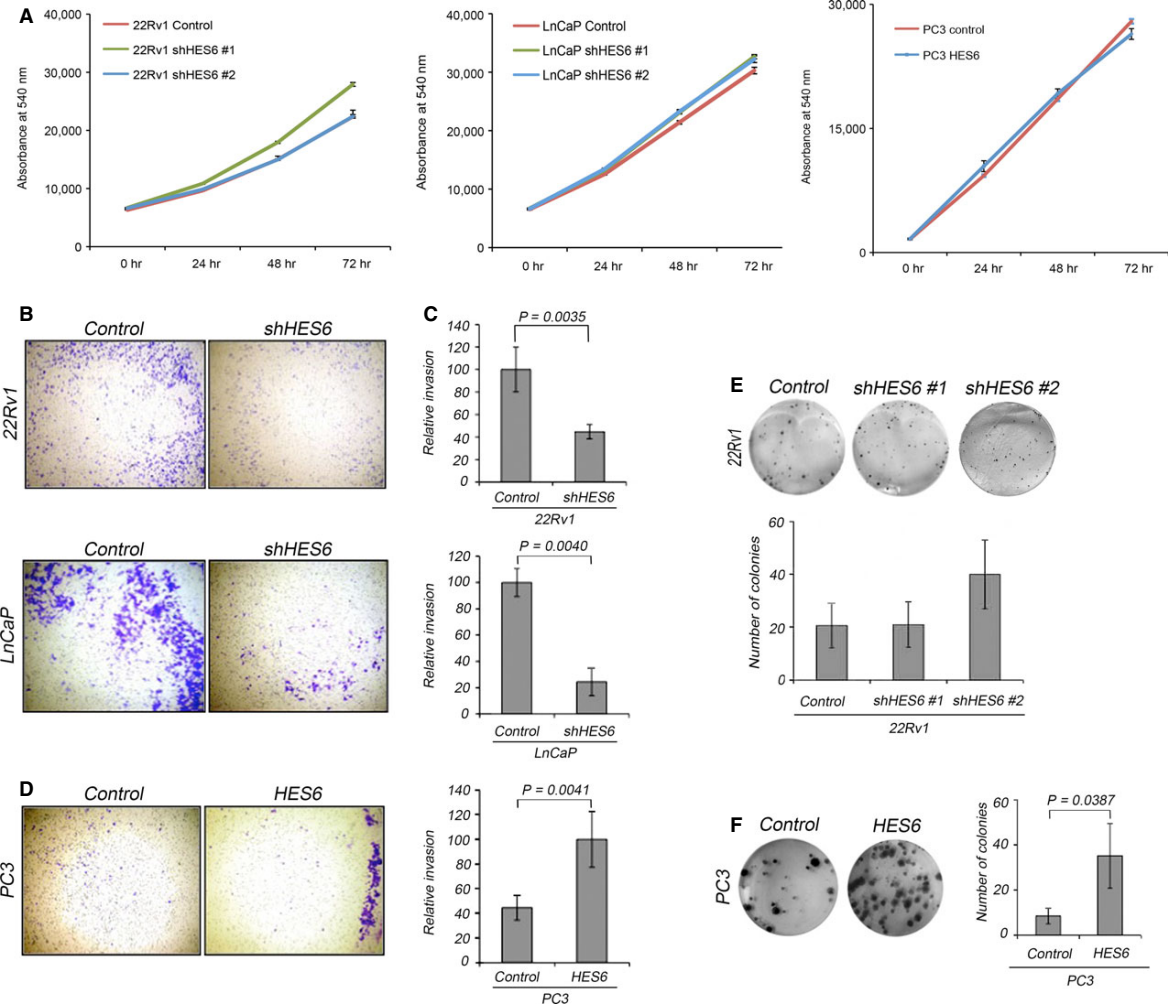
HES6 promotes cancer cell invasion and colony formation. (A) AlamarBlue proliferation assays following HES6 shRNA knockdown in 22Rv1 and LnCaP (green and blue lines in left and middle panels), and HES6 overexpression in PC3 (blue line in right panel). Growth curves show that neither knockdown nor overexpression of HES6 affected growth. (B and C) Representative images showing 22Rv1 and LnCaP cells that invaded through a Matrigel membrane (B). Both cell lines showed a significant decrease in invasiveness (C) after HES6 shRNA knockdown. Quantitative data are averaged across three independent experiments. Values are expressed as mean ± SEM. (D) Representative images and quantification of HES6-overexpressing PC3 cells during invasion of a matrigel membrane. Values are expressed as mean ± SEM. (E and F) Representative pictures and quantification of colony-formation assays for 22Rv1 cells in which HES6 expression is knocked down (E) and in PC3 cells overexpressing HES6 (F). The quantitative data are averaged across two independent experiments. Values are expressed as mean ± SEM.

To test this idea, we assayed the effects of up- and down-regulating HES6 expression on standard growth in culture, clonogenic growth, cell migration and invasion. Some of these *in vitro* cellular behaviours, including clonogenic growth and invasion through various matrices, correlate with the ability to metastasize *in vivo* 38,39. For the clonogenic and growth assays, we tested a second shRNA and found similar results, confirming that when seen, effects of knock down were attributable to HES6 suppression rather than off-target effects. Using lentiviral plasmid transfer, we reversed baseline levels of HES6 (Fig.2A) by stably engineering its expression into PC3 cells and knocking down its expression in 22Rv1 and LnCaP cells (Fig. S5). Whereas HES6 overexpression or knockdown did not have a noticeable effect on cell viability or growth rate (Fig.5A), HES6 levels significantly affected the ability of cancer cells to invade. HES6 knockdown in 22Rv1 and LnCaP significantly impaired cell invasion through a matrigel membrane, and PC3 cells overexpressing HES6 showed a significant increase in invasive behaviour compared to controls (Fig.6B–D). In line with these observations, HES6 affects the clonogenic potential of cancer cells. HES6 knockdown in 22Rv1 and LnCaP cell did not significantly decrease the number of colonies, but HES6 overexpression in PC3 increased the capacity of the cells to form colonies fourfold (Fig.6E and F). As with previous studies that showed HES6 expression distinguishes metastatic from non-metastatic prostate cancers 6,40, these results strongly suggest that HES6 imbues cancer cells with the capacity to escape the prostate and generate metastatic clones.

### HES6 controls a number of pathways that contribute to cancer progression

**Figure 7:**
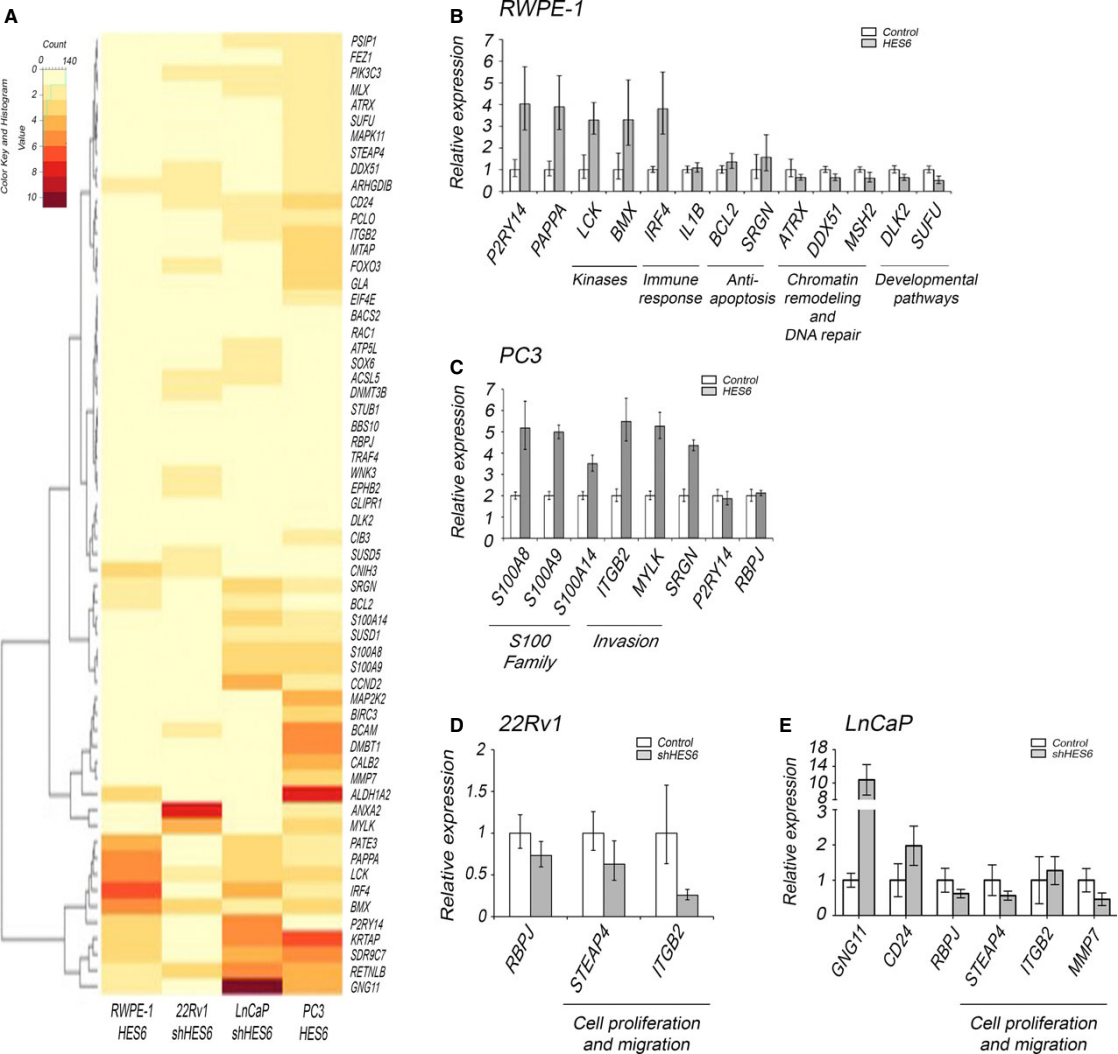
HES6 controls different pathways in benign and cancer cell lines. (A) Heat map showing changes in gene expression associated with HES6 overexpression in RWPE-1 and PC3 cell lines and with HES6 knockdown in 22Rv1 and LnCaP cells. (B–E) qPCR analysis of differentially expressed transcripts after HES6 overexpression in RWPE-1 (B) and PC3 (C) and after HES6 knockdown in 22Rv1 (D) and LnCaP (E). Note invasion genes affected in all cancer cell lines, *i.e*. ITGB2 in PC3 and 22Rv1 and MMP7 in LnCaP. Values represent three technical replicates, mean ± SEM.

To better understand HES6’s role in cancer progression, we analysed global gene expression in cells engineered to overexpress or knock down HES6 and compared the data sets to identify genes differentially expressed in the two cell types. Despite previous indicators that HES6 might down-regulate Notch signalling, we saw no evidence that HES6 overexpression significantly modulated any Notch target (Fig.7A). However, it did modulate the expression of genes involved in a number of other pathways. Intriguingly, these HES6-regulated pathways differed in benign cells compared to malignant cells (Fig.7A). In benign cells, overexpressed HES6 induced IRF4, LCK and BMX—genes involved in immunosurveillance and cell survival (Fig.7B). Two other genes previously implicated in lung cancer, P2RY14 and PAPPA 41,42, were also expressed at significantly higher levels when HES6 was overexpressed. Furthermore, in benign RWPE-1 cells, HES6 overexpression significantly down-regulated ATRX and DDX51 genes, which encode proteins involved in chromatin remodelling and telomere lengthening. Interestingly, we found HES6 overexpression also down-regulated DLK2, a gene previously implicated in prostate tumourigenesis 43 whose protein product inhibits Notch signalling. Also down-regulated was suppressor of fused (SUFU), which is considered a tumour suppressor gene because of its inhibitory influence on the hedgehog pathway (Fig.7B). Hedgehog signalling has been implicated in prostate cancer development, metastasis and androgen-independent growth 44,45, so repression of SUFU by HES6 could represent a new mechanism whereby prostate cancer cells induce hedgehog signalling to promote aggressive behaviours.

PC3 prostate cancer cells have relatively low basal levels of HES6. Engineered overexpression of HES6 in PC3 cells most significantly induced genes involved in cell structure, motility, invasion and metastasis (Fig.7C). Significantly overexpressed genes included three members of the S100 family—S100A8, S100A9 and S100A14—as well as serglycin (SRGN), myosin light chain kinase (MYLK), and integrin beta 2 (ITGB2). These genes were previously implicated in progression of several tumour types, including prostate cancer: S100 proteins are overexpressed in prostate cancer and can be detected in circulating tumour cells 46,47; SRGN promoted metastasis in nasopharyngeal carcinoma 48; MYLK was identified in a microarray study as one of the most discriminative genes between normal and malignant prostate cells 49; and integrin beta 2, as well as other integrins, are essential for different types of cancer cells to interact with the extracellular matrix and trigger intracellular signals to support survival and migration 50.

LnCaP and 22Rv1 prostate cancer cells have higher basal levels of HES6 expression than PC3 (Fig.2A), so we used HES6 knockdown in these cells to identify HES6 targets. In these cell lines, HES6 regulated a set of genes that only partially overlapped with those found in PC3 cells, but the most highly affected pathways in all cancer cell lines were strikingly similar. Knockdown studies in LnCaP and 22Rv1 revealed a role for HES6 in regulating genes involved in migration and invasion, as seen in PC3. HES6 knockdown influenced the expression of two cell migration-associated genes—metalloproteinase 7 (MMP7) and ITGB2 (Fig.7D and E)—as well as STEAP4, which was previously implicated in prostate cancer cellular proliferation 51. In LnCaP cells, HES6 knockdown significantly induced expression of the G-protein GNG11, which not only induces cell senescence 52 but also exhibits down-regulation in splenic marginal zone lymphoma 53. HES6 down-regulation in LnCaP significantly increased expression of CD24 (Fig.7E)—a cell surface protein that plays important roles in cell–cell and cell-extracellular matrix interactions. Some studies have shown that CD24 is highly expressed in high-grade prostate cancers 54. However, progenitor cancer cells with the ability to initiate new tumours express lower levels of CD24 than differentiated cells 55. In this context, HES6 may enhance tumour initiation by supporting expression of CD24.

## Discussion

Our results confirm differential expression of Notch pathway members in aggressive prostate cancer and indicate a role for HES6, either by direct action or by activation of other pathways, in tumour progression. Our data also suggest that expression of Notch pathway members and of HES6 may be useful in distinguishing indolent from lethal prostate cancers.

Globally, Notch receptor expression and Notch signalling activation were inversely correlated with malignant behaviours of cancer cells. We found that prostate benign cells generally have higher baseline Notch1 and Notch2 expression and more Notch pathway activation than cancer cells. Notch pathway blockade—a proposed therapeutic modality for breast cancer 56—had no effect on prostate cancer viability. To the extent that *in vitro* assays reflect cancer biology *in vivo*, these results suggest that Notch blockade may not be therapeutically helpful in prostate cancer patients.

Our study of Notch signalling dynamics was limited to signalling within epithelial and cancer cells. It is possible that Notch signalling participates in important interactions between stroma and prostate epithelium and/or cancer. Indeed, Orr *et al*. have demonstrated that the Notch inhibitor DLK elicits growth inhibitory effects on prostate epithelial cells indirectly through an unknown mechanism 57. Our results showing higher expression and activity of the Notch pathway in benign *versus* malignant prostate cells combined with our findings after up- and down-regulating Notch signalling in prostate cells suggest that growth suppression by DLK would most likely require engagement of alternate pathways (other than Notch) within prostate epithelial cells. A potential role of Notch signalling in the prostate echoes findings in drosophila gut wherein Notch ligands expressed in stem cells drive differentiation and proliferation in adjacent epithelial progeny through induction of HES/HEY family members 58. In the prostate, basal cells may use JAG1 and DLL1 to engage NOTCH3 on luminal cells to stimulate proliferation and differentiation. Prostate cancers, given their universally luminal differentiation, may respond to Notch signalling in a similar manner to benign luminal cells. In support of this model, we found that benign basal cells (PrEC) co-expressed relatively high levels of Notch ligands JAG1 and DLL1 (Fig.1), and particularly low levels of Notch3 receptor (Figs1 and 2). In contrast Notch3 is elevated in benign lumina cells (RWPE) and malignant (LnCaP, PC3 and 22RV1) lines (Fig.2). We should note, however, when cultured alone, Notch signalling did not affect proliferation in our studies. To the extent that it affects cell growth, it is likely that Notch signalling requires mixtures of different cell types.

HES6 is a regulator of cell fate, either in normal development during neurogenesis and myogenesis 59,60, and establishment of neuroendocrine phenotype in prostate cancer 37. Neuroendocrine cells show a decrease in AR signalling, express neuronal markers and have stem cell characteristics 61. Therefore, HES6 may play an important role in the plasticity of prostate cancer cells.

HES6, though homologous to Notch pathway targets, was unresponsive to NOTCH3–the Notch receptor most highly expressed in prostate cancer cells. HES6 facilitated colony formation, invasion and migration, perhaps through its effects on relevant cell pathways such as CD24 and integrin signalling. We found a significant decrease in HES6 expression in androgen-deprived cell lines and in prostate cancer specimens from patients treated with ADT. However, in a variable time frame, prostate cancer becomes resistant to ADT, and cancer cells are able to reactivate and AR signalling. It was previously shown that Notch signalling targets, such as HEY1, can bind AR and suppress androgen signalling 62. Moreover, AR binds upstream of HES6 coding sequence to upregulate HES6 mRNA levels 40 reinforcing a feedback regulation between androgens and Notch pathway. Our results show that DHT induces an up-regulation of HES1, HEY1 and HES6. We therefore speculate that androgens enhance Notch signalling effects and the activity of these HES family proteins as part of prostate cancer progression.

Our results show that HES6 reflects and mediates an aggressive phenotype in hormone-naïve prostate cancers. As such, measuring levels of HES6 may help distinguish indolent from aggressive cancers, as suggested previously 6. The identification of the invasive and clonogenic advantages conferred by HES6 to cancer cells and the discovery of other cellular pathways that HES6 activates may reveal new ways to interfere with prostate cancer progression—discoveries that could ultimately lead to the development of novel therapies for men with aggressive prostate tumours.

## Supporting Information

**Figure S1** qRT-PCR analysis for *NOTCH3* expression in prostate cell lines. Error bars, mean ± SEM of three technical replicates.

**Figure S2** qPCR analysis of Notch pathway members’ expression in cancer cell lines relative to PrEC. The exclusion of RWPE-1 from the analysis highlights a significant increase in *NOTCH3* in cancer cells.

**Figure S3** qPCR shows increase in *NOTCH3* transcripts after overexpression of NICD3. We used TaqMan primers to detect NICD3 transcripts because the antibody used to detect NOTCH3 protein expression in Western blots (Pro2311; Cell Signaling) recognizes the transmembrane domain and is unable to detect the intracellular portion of the receptor.

**Figure S4** qPCR shows *PSA* transcript levels at several time-points after DHT stimulation of LnCaP cells.

**Figure S5** Manipulation of HES6 protein levels in cancer cell lines.

**Table S1** Gene names and corresponding TaqMan probes used in gene expression studies.

**Table S2** siRNA sequences for Notch3 knockdown in prostate cancer cells.
