# Supplementary figures and images for: HES6 promotes prostate cancer aggressiveness independently of Notch signalling
Source: J Cell Mol Med. 2015 Apr 12;19(7):1624–36. doi: 10.1111/jcmm.12537 (PMC4511360; doi:10.1111/jcmm.12537)

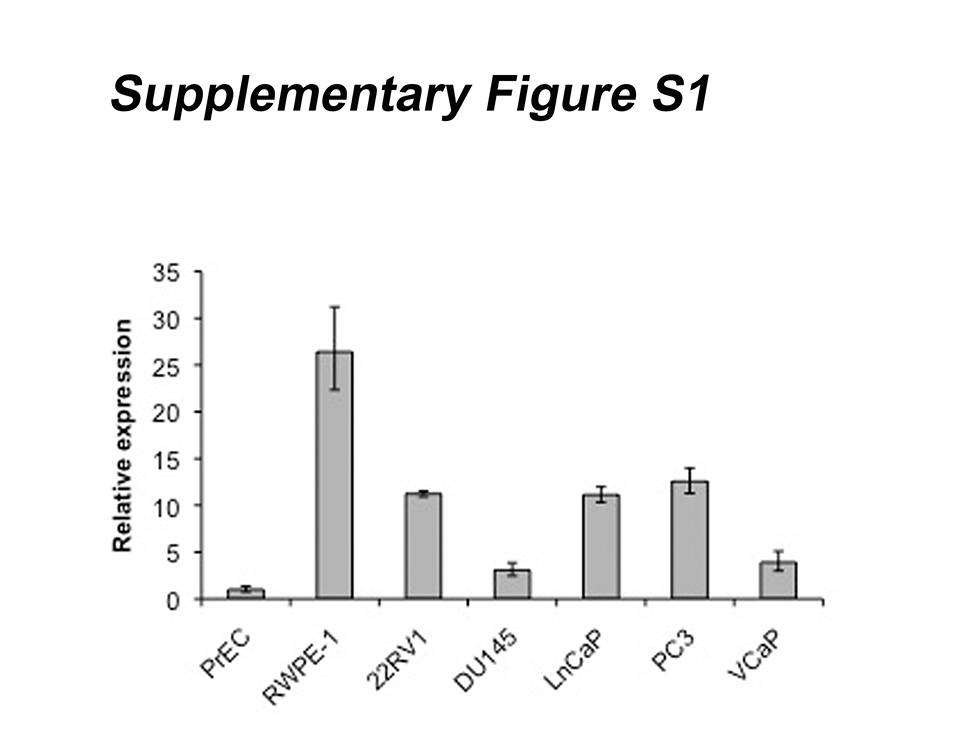

Supplement: Supplementary file 1 [file jcmm0019-1624-sd1.tif]

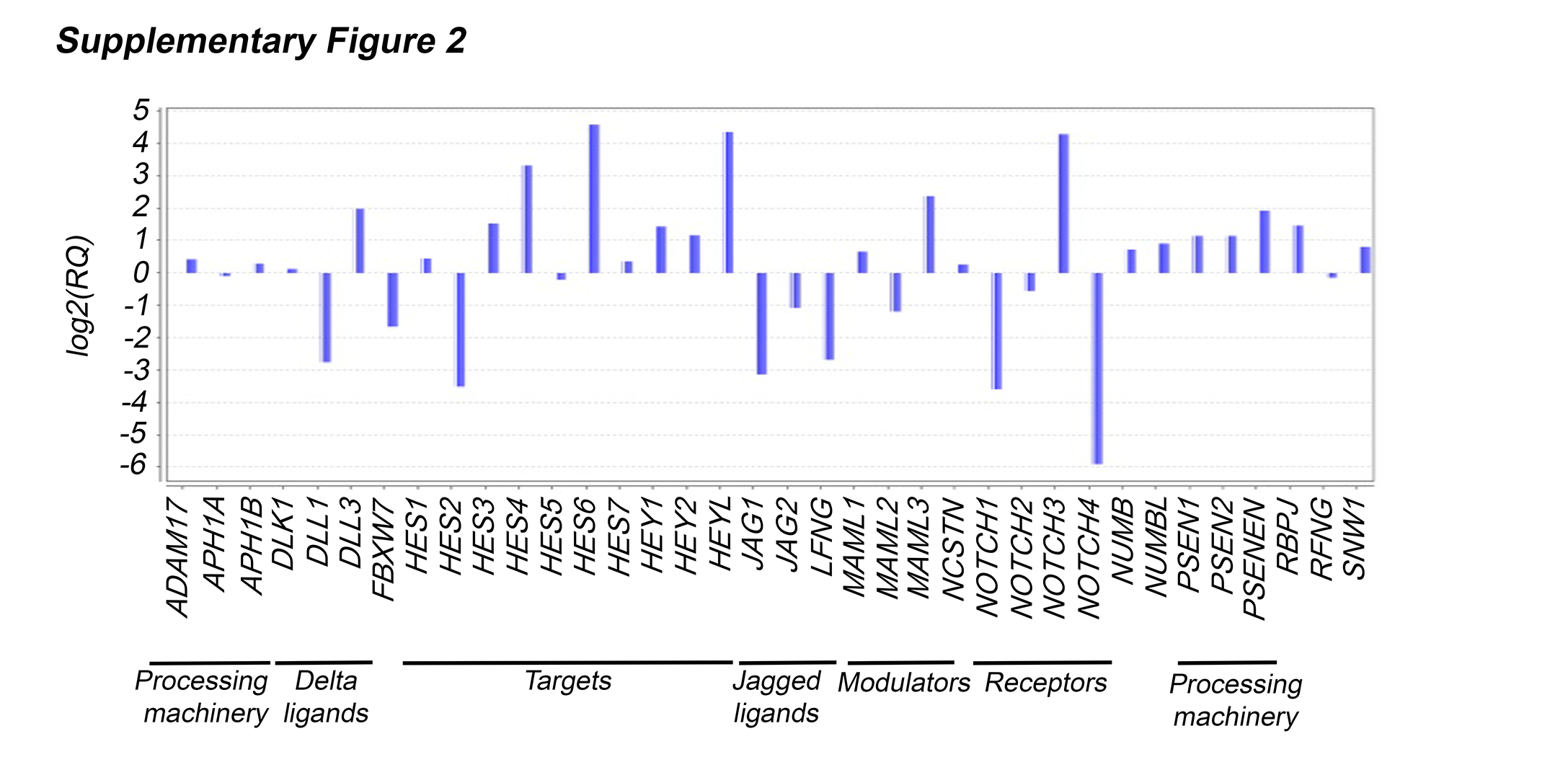

Supplement: Supplementary file 2 [file jcmm0019-1624-sd2.tif]

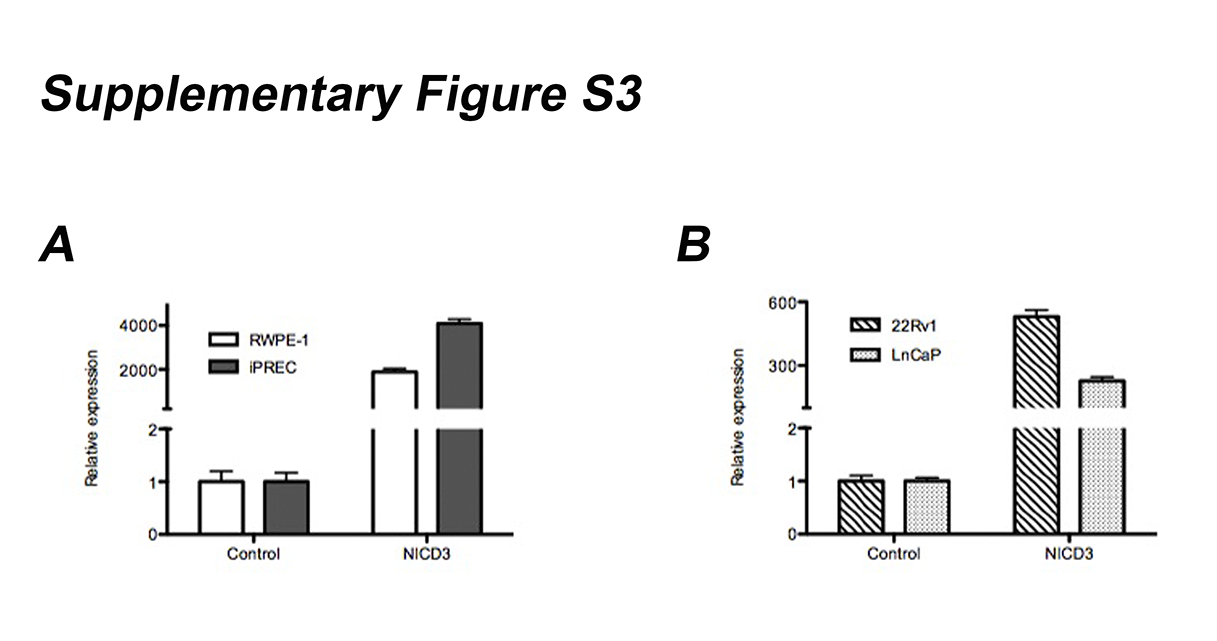

Supplement: Supplementary file 3 [file jcmm0019-1624-sd3.tif]

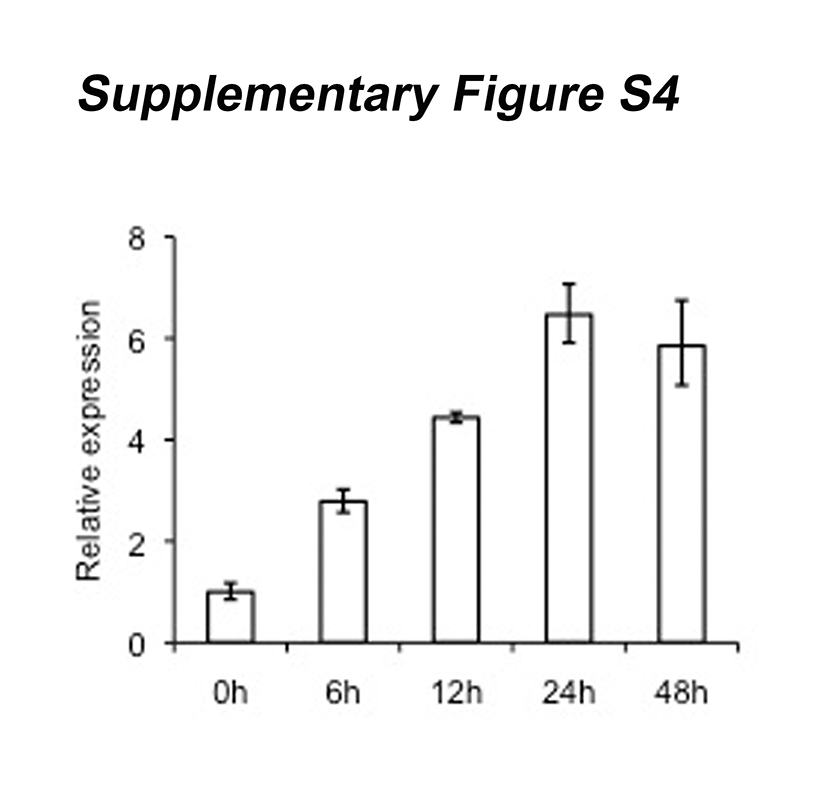

Supplement: Supplementary file 4 [file jcmm0019-1624-sd4.tif]

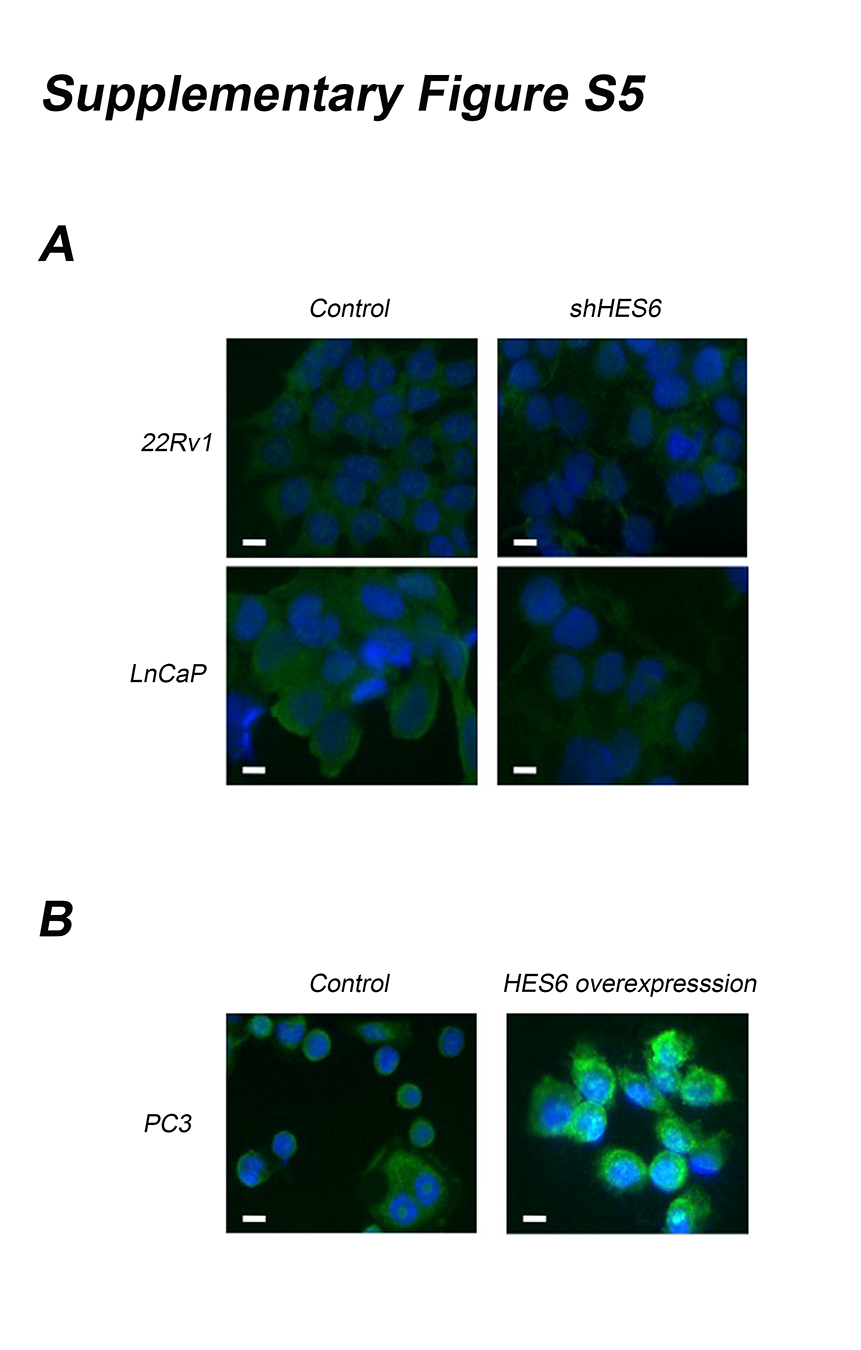

Supplement: Supplementary file 5 [file jcmm0019-1624-sd5.tif]

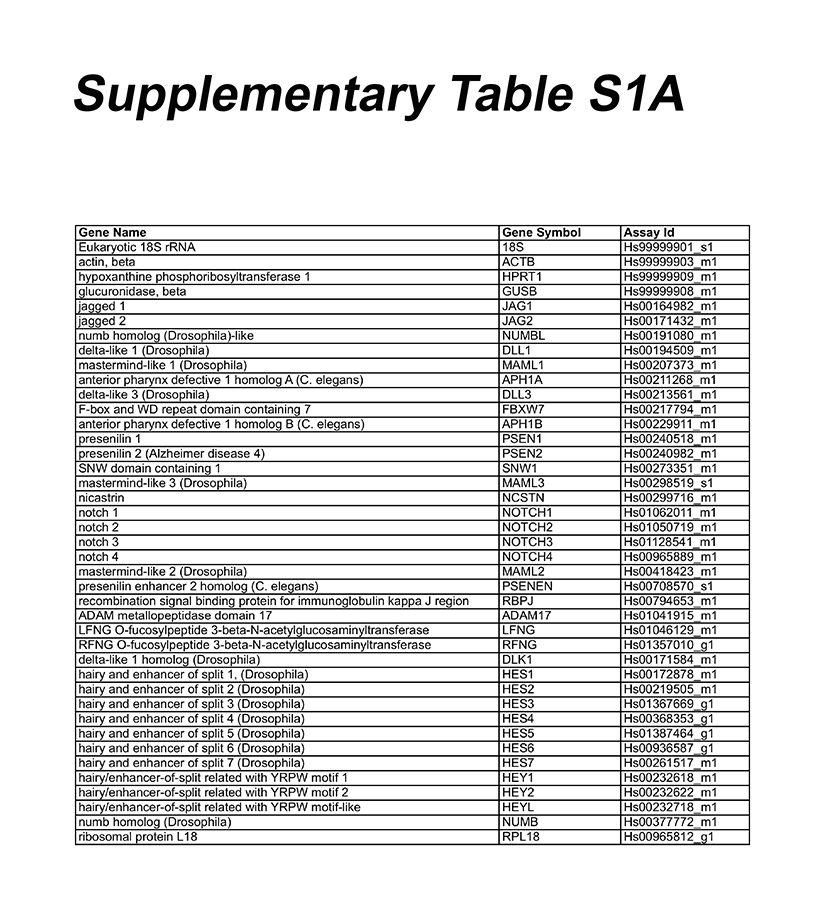

Supplement: Supplementary file 6 [file jcmm0019-1624-sd6.tif]
